# Indole-3-Acetic Acid Produced by *Burkholderia heleia* Acts as a Phenylacetic Acid Antagonist to Disrupt Tropolone Biosynthesis in *Burkholderia plantarii*

**DOI:** 10.1038/srep22596

**Published:** 2016-03-03

**Authors:** Mengcen Wang, Seiji Tachibana, Yuta Murai, Li Li, Sharon Yu Ling Lau, Mengchao Cao, Guonian Zhu, Makoto Hashimoto, Yasuyuki Hashidoko

**Affiliations:** 1 Research Faculty of Agriculture, Hokkaido University, Kita 9, Nishi 9, Kita-ku, Sapporo 060-8589, Japan; 2 Institute of Pesticide and Environmental Toxicology, Zhejiang University, No. 268 Kaixuan Road, Hangzhou 310029, China; 3 Frontier Research Center for Post-Genome Science and Technology, Faculty of Advanced Life Sciences, Hokkaido University, Kita 10, Nishi 8, Kita-ku, Sapporo 060-0810, Japan

## Abstract

*Burkholderia heleia* PAK1-2 is a potent biocontrol agent isolated from rice rhizosphere, as it prevents bacterial rice seedling blight disease caused by *Burkholderia plantarii*. Here, we isolated a non-antibacterial metabolite from the culture fluid of *B. heleia* PAK1-2 that was able to suppress *B. plantarii* virulence and subsequently identified as indole-3-acetic acid (IAA). IAA suppressed the production of tropolone in *B. plantarii* in a dose-dependent manner without any antibacterial and quorum quenching activity, suggesting that IAA inhibited steps of tropolone biosynthesis. Consistent with this, supplementing cultures of *B. plantarii* with either L-[*ring*-^2^H_5_]phenylalanine or [*ring*-^2^H_2~5_]phenylacetic acid revealed that phenylacetic acid (PAA), which is the dominant metabolite during the early growth stage, is a direct precursor of tropolone. Exposure of *B. plantarii* to IAA suppressed production of both PAA and tropolone. These data particularly showed that IAA produced by *B. heleia* PAK1-2 disrupts tropolone production during bioconversion of PAA to tropolone via the ring-rearrangement on the phenyl group of the precursor to attenuate the virulence of *B. plantarii. B. heleia* PAK1-2 is thus likely a microbial community coordinating bacterium in rhizosphere ecosystems, which never eliminates phytopathogens but only represses production of phytotoxins or bacteriocidal substances.

Troponoids are a unique group of natural products containing a seven-membered aromatic ring with various substitutions^1^,^2^,^3^,^4^. These compounds have various bioactivities, including antiviral, antitumor, antioxidant, anti-inflammatory, and insecticidal effects^4^. Tropolone, which is produced by *Burkholderia plantarii*, is the phytotoxin responsible for rice seedling blight^5^. This was demonstrated when treatment only with exogenous tropolone was able to phenocopy *B. plantarii* infestation, which manifests as chlorosis, shoot-stunting, and root inhibition^6^,^7^.

The potent iron-chelating property of tropolone not only accounts for its broad-spectrum antimicrobial activity against bacteria and fungi^8^ but also contributes to its virulence and the symptoms associated with the onset of bacterial rice seedling blight^6^,^9^. When this phytotoxin accumulates in culture fluid, or is exogenously added to the culture medium, it triggers production of extracellular polysaccharide by bacteria^10^. Despite its key role in virulence, the tropolone biosynthetic pathway in *B. plantarii* remains unclear. In phytopathogenic eubacteria, production of virulence factors is often under the control of a sophisticated regulatory system^11^,^12^,^13^,^14^. In several human-pathogenic *Burkholderia* species, virulence factors, including capsular polysaccharide I^15^, helicase inhibitor^16^, and actin polymerization *bimABm* gene^17^ are regulated by quorum-sensing molecules^18^,^19^,^20^.

We previously performed a screen to identify tropolone- and catechol-tolerant microbial isolates^7^. Here, we screened 15 of these isolates for their efficacy as biocontrol agents against *B. plantarii*. One of these isolates, *Burkholderia heleia* PAK1-2, suppressed blight symptoms when topically applied to rice seedlings. A thin-layer chromatography and NMR analysis-guided bioassay that monitors tropolone production identified a non-antibacterial metabolite, indole-3-acetic acid (IAA), that was produced by *B. heleia* PAK1-2 and alleviated *B. plantarii* virulence. We show that IAA inhibits the metabolism of an intermediary compound in tropolone biosynthesis to prevent the ring re-arrangement required for the conversion of phenylacetic acid (PAA) to tropolone.

## Results

### *Burkholderia heleia* PAK1-2 is a potent biocontrol agent that blocks *B. plantarii*-dependent rice seedling blight

We determined that PAK1-2 was an irregular rod-shaped gram-negative bacterium of class *Betaproteobacteria*; alignment of its 1.5 kbp 16S rRNA gene sequence revealed homology with *Burkholderia* species (accession no. AB787501). Phylogenetic analysis for the species identification was done using multiple-aligned sequences of *Burkholderia* sp. PAK1-2 and other *Burkholderia* species including some type-strains (Figure S1). Accordingly, PAK1-2 formed a small clade together with another *Burkholderia heleia* strain (NBRC 101817^T^)^21^ in the phylogenetic cluster of genus *Burkholderia*. This allowed us to positively identify PAK1-2 as a *B. heleia* strain.

**Figure 1:**
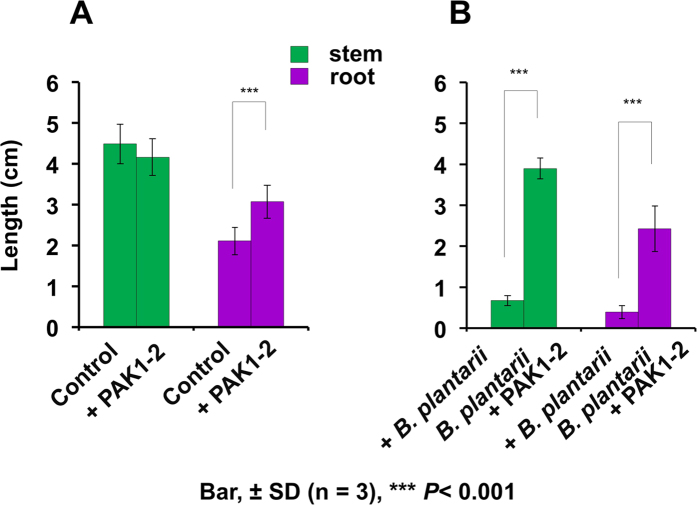
Biocontrol efficacy of *B. heleia* PAK1-2 against *B. plantarii*-caused rice seedling blight. ‘Control’ is the rice seedlings without any inoculation; ‘+PAK1-2’ indicates the rice seedlings inoculated with *B. heleia* PAK1-2 only (**A**). Same as A, ‘+*B. plantarii*’ indicates rice seedlings inoculated with *B. plantarii* only, and ‘*B. plantarii* +PAK1-2’ is the rice seedlings inoculated with both *B. plantarii* and *B. heleia* PAK1-2. Values (cm) are means ± SD (shown by error bars) (*n* = 30). ***P < 0.001 as determined by Student’s *t-*test.

In the dual culture system, point-inoculated *B. heleia* PAK1-2 showed a weak growth-inhibitory activity against *B. plantarii* (Figure S2). In addition, *B. heleia* PAK1-2 significantly repressed the symptoms of blight on rice seedlings infested with *B. plantarii.* The effective biocontrol properties of *B. heleia* PAK1-2 *in vivo* were observable both in the shoots and in the roots (Fig. 1). In particular, rice seedlings inoculated with *B. heleia* PAK1-2 had accelerated root growth and lateral root development even in the absence of *B. plantarii* infection.

### Inhibition of tropolone biosynthesis by IAA produced by *B. heleia* PAK1-2

**Figure 2:**
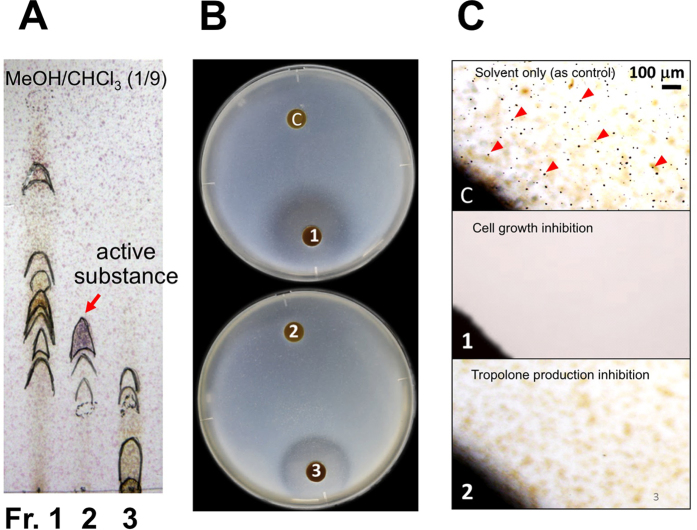
Tropolone production and cell growth of *B. plantarii* exposed to fractionated metabolites from *B. heleia* PAK1-2 or to IAA-supplemented medium. Fractions showing a similar pattern on silica gel thin-layer chromatography were combined to give three fractions (5–20%, 25–50%, and 60–100% MeOH/chloroform for Fr. 1, 2, and 3, respectively) (**A**). Semi-quantitative analysis of tropolone production and *B. plantarii* growth after exposure to fractionated metabolites from *B. heleia* PAK1-2 loaded on paper discs (**B**). Microscopic observation of tropolone production by *B. plantarii* in the area around the paper disc, in which fraction 2 (100 μg disc^−1^) had been loaded, while cell growth and tropolone production were eliminated in the area around the paper disc loaded with fraction 3 (100 μg disc^−1^) (**C**). Red arrow in C indicates the typical tropolone-iron crystals, whereas the greyish mottled objects in the background are the grown *B. plantarii* colonies from *B. heleia*-impregnated bacterial cells.

To isolate the active substance produced by *B. heleia* PAK1-2 capable of inducing *B. plantarii* cellular responses, we performed semi-quantification of tropolone production in *B. plantarii* that was exposed to fractionated metabolites of *B. heleia* PAK1-2. In contrast to untreated cells, production of tropolone by *B. plantarii* was repressed by fractions obtained by silica gel column chromatography (see Materials and Methods) at 100 μg disc^−1^ (Fig. 2A), and we established that this was linked to repression of cell growth (Fig. 2B,C). However, we were unable to obtain reproducible production of any growth-inhibitory compounds from large-scale cultures of *B. heleia*. By contrast, fraction 2 did not repress *B. plantarii* growth, but retained the ability to inhibit tropolone production (Fig. 2C). The active chemical compound in fraction 2 was subsequently identified as IAA (Table S1). In support of this, tropolone production was inhibited in monocultures of *B. plantarii* directly supplemented with exogenous IAA; this occurred in the absence of effects on cell growth (Fig. 2B).

Tropolone production of *B. plantarii* is under the control of the *N*-acyl-L-homoserine lactone (AHL)-QS regulatory system^10^. Therefore, we evaluated whether IAA-dependent repression of tropolone production was due to its modulation of the *B. plantarii* AHL-QS-associated genes, *plaI* and *plaR* (Figure S3). Because IAA did not elicit any statistically significant repression of both *plaI* and *plaR* transcription, it was concluded that IAA does not disrupt AHL-QS in *B. plantarii.*

### Identification of phenyl acetic acid produced by *B. plantarii* at the early growth stage

*B. plantarii* shake-cultured for 24 h yielded a mixture of ethyl acetate (EtOAc)-soluble secondary metabolites, which were subjected to ^1^H-NMR and MS analyses for metabolic profiling as described by Clarke and Haselden^22^. In direct electron impact ionization (EI)-MS, tropylium cation (*m*/*z* 91) appeared as the base fragment and the parent ion was predicted at *m*/*z* 136 (Figure S4A), whereas direct field ionization (FI)-MS gave a molecular ion (M^+^) peak at *m*/*z* 136 as the base peak (Figure S4B). In GC-MS analysis of the metabolite mixture, the most dominant peak was detected at *t*R 4.19 min in the total ion chromatographic profile (Figure S4C). For this main peak, EI for GC-MS showed a molecular ion at *m*/*z* 136 ([M]^+^, 31%) and tropylium cation at *m*/*z* 91 ([M−COOH]^+^, 100%) (Figure S4D) consistent with phenylacetic acid (PAA) reported by Kim *et al.*^23^ (2004). In addition, the dominant compound gave the molecular ion ([M]^+^) peak at *m*/*z* 136 by GC-MS under FI mode (Figure S4E). ^1^H-NMR spectrum of the EtOAc soluble mixture also showed aromatic proton and equivalent methylene proton signals, assignable to the phenyl group and the C2 methylene moiety of PAA (Figure S5). The most dominant metabolite produced at the early culture stage was thus identified as PAA.

### Linkage of tropolone biosynthesis to accumulation of phenylacetic acid

**Figure 3:**
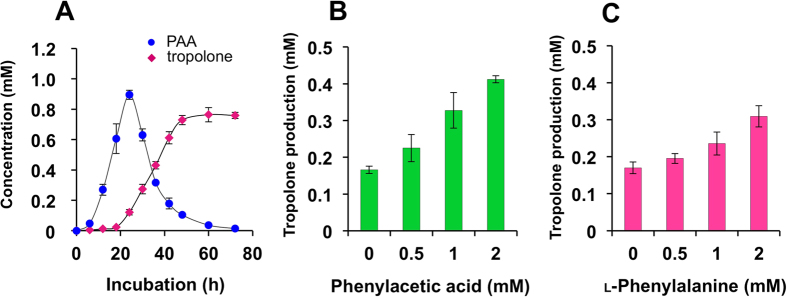
Relationship between tropolone production and dose of phenylacetic acid or L-phenylalanine in *B. plantarii*. Kinetics of tropolone and PAA production in *B. plantarii* (**A**). Dose-dependent promotion of tropolone production in *B. plantarii* by addition of exogenous PAA at a concentration range of 0.5–2 mM (**B**). Dose-dependent promotion of tropolone production in *B. plantarii* by addition of L-phenylalanine at a concentration range of 0.5–2 mM (**C**). Values are means ± SD (shown by error bars) (*n* = 3).

GC-MS and NMR-based metabolic profiling showed that, before the appearance of tropolone, PAA was the major metabolite produced by *B. plantarii* during the exponential phase (Fig. 3A). Further quantitative analysis for PAA in the medium supplemented with L-phenylalanine revealed that PAA production by *B. plantarii* was sustained over 24 h, where it reached a maximum concentration of 895.3 μM. However, PAA levels decreased significantly in cultures beyond 24 h and fell to 2% (13.2 μM) of the maximum by 72 h. Concomitant with the rapid decrease of PAA, tropolone production accelerated robustly beginning at 24 h and reached its maximum level (763.9 μM) at 60 h (Fig. 3A). Tropolone production dose-dependently increased when *B. plantarii* was fed with 0.5–2 mM PAA (Fig. 3B). Together, these qualitative and quantitative data reveal a close link between upstream PAA production and downstream tropolone production, and strongly suggest that PAA is the direct precursor of tropolone.

PAA can be derived from shikimic acid or via an oxidative decarboxylation-coupled deamination of L-phenylalanine. Therefore, conversion of L-phenylalanine to tropolone via PAA by *B. plantarii* was also confirmed by supplementing medium containing the phytopathogen with L-phenylalanine instead of PAA (Fig. 3C). However, PAA induced greater tropolone production than that observed with L-phenylalanine at the same concentration (2 mM).

**Figure 4:**
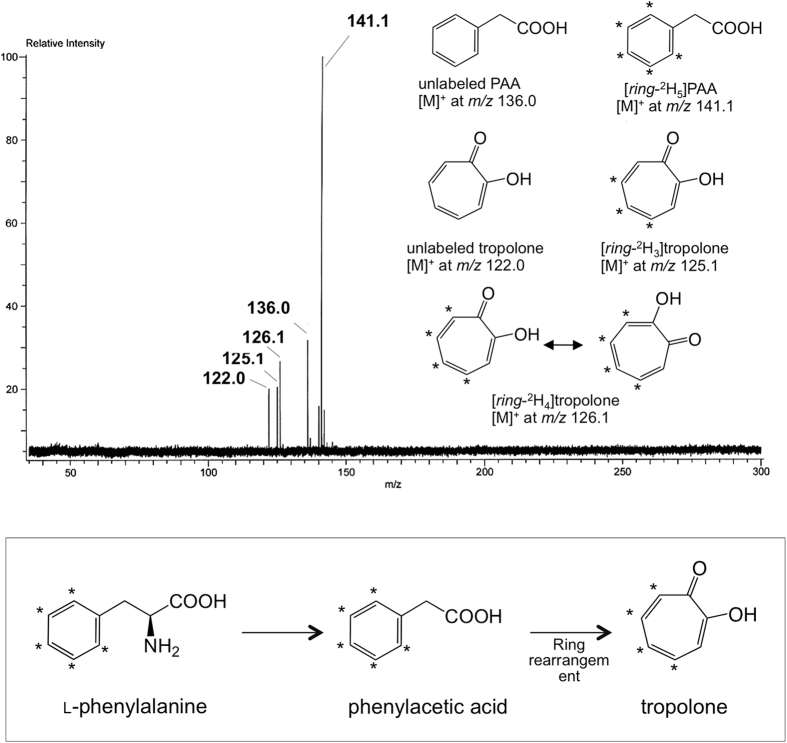
Identification of isotopomeric tropolones and phenylacetic acids from L-[*ring*-^2^H_5_] phenylalanine. FI-MS spectrum of metabolites corresponding to PAA and tropolone obtained from *B. plantarii* supplemented with L-[*ring*-^2^H_5_]phenylalanine as shown in the top panel. The structures of tropolone, PAA and their isotopomers deduced from the molecular weights are also shown in the same panel. In the conversion of L-[*ring*-^2^H_5_]phenylalanine into [*ring*-^2^H_4_]tropolone via [*ring*-^2^H_5_]PAA, one deuterium at the C-2 or C-6 position was replaced with a proton during the ring rearrangement (bottom panel). Asterisk indicates the position of the deuterated carbon.

After growth in PD broth media supplemented with L-phenylalanine-[*ring*-^2^H_5_] for 36 h, the PAA produced was extracted from the acidified culture fluid. PAA-[*ring*-^2^H_5_] from labelled L-phenylalanine and non-deuterated PAA from L-phenylalanine or shikimate originally in the medium or biosynthesized by *B. plantarii* from ingredients in the plain medium were present in a ratio of 3.6:1 (calculated from the relative peak intensity in FI-MS spectrum) (Fig. 4). The incorporation rate of L-[*ring*-^2^H_5_]phenylalanine into PAA was 78.3%. Further incorporation of deuterium atoms of [*ring*-^2^H_5_]PAA into tropolone led to tropolone-*d*_4_ and tropolone-*d*_3_ with a ratio of 1.6:1. These data are consistent with PAA being the direct precursor of tropolone.

Treatment of PAA with TfOD yielded a mixture of deuterated PAA with 2- and 5-substituted deuterium atoms on the benzene ring. GC-MS/MS analysis revealed that a mixture of [*ring*-^2^H_2_-_5_]PAA was converted by *B. plantarii* to tropolone-*d*_2~4_ (Figure S6). This experiment provided evidence that PAA is the direct precursor of tropolone in *B. plantarii*. The absence of one deuterium on the labelled tropolone molecule may indicate that the benzene ring is rearranged concomitantly with an aromatic proton exchange.

### Interference of the tropolone biosynthetic pathway of *B. plantarii* by indole-3-acetic acid and its analogous compounds

**Figure 5:**
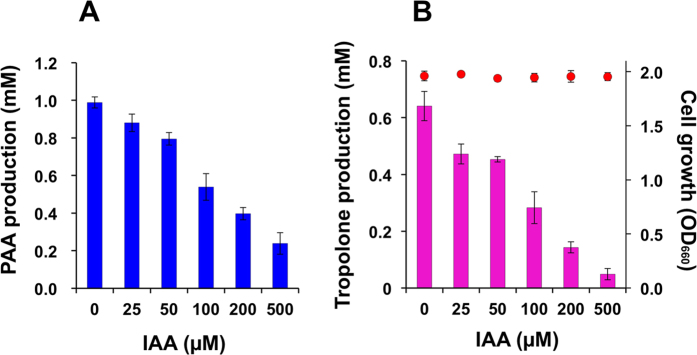
Effect of indole-3-acetic acid on PAA and tropolone production by *B. plantarii*. Production of both PAA (**A**) and tropolone (**B**) was quantified from *B. plantarii* PD broth cultures containing 25–500 μM IAA. Values are the mean ± SD (shown as error bars) (*n* = 3). The cell growth of each shake culture was also monitored based on the optical density at 660 nm (shown by red circles in panel B).

In the presence of 500 μM IAA, PAA levels decreased from 980 μM to 240 μM, representing 76% repression of PAA production (LC_50_ = *ca.* 125 μM) (Fig. 5A). IAA likely prevents the metabolism of L-phenylalanine, but it is also likely that the suppression of decarboxylation-coupled deamination on L-phenylalanine by IAA is not the main mode of action in the inhibition of tropolone production by *B. plantarii*. Conversely, 500 μM IAA prevented 92% of tropolone production (LC_50_ = *ca.* 90 μM), mainly from PAA, showing that the repressive effect of IAA against tropolone production is mediated through inhibition of the rearrangement of the benzene ring of PAA into a seven-membered tropolone ring (Fig. 5B).

Neither indole nor a series of the other indolic analogues tested suppressed PAA production by *B. plantarii* cells, whereas tropolone production was slightly repressed upon exposure to the indole derivatives, except for IAA. IAA reduced tropolone levels by two fold. Cell growth of *B. plantarii* was unaffected by the indole derivatives, including IAA (Figure S7).

### Interference of the tropolone biosynthetic pathway of *B. plantarii* by PAA analogues

**Figure 6:**
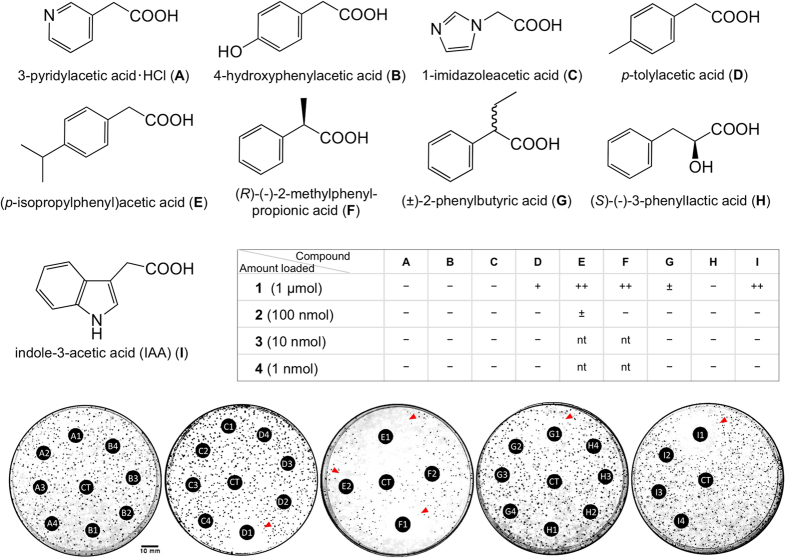
Effect of compounds structurally similar to PAA on production of tropolone by *B. plantarii*. Eight compounds, i.e., 3-pyridylacetic acid HCl (**A**), 4-hydroxyphenylacetic acid (**B**), 1-imidazoleacetic acid (**C**), *p*-tolylacetic acid (**D**), (*p*-isopropylphenyl)acetic acid (**E**), (*R*)-(–)-2-methylphenylpropionic acid (**F**), (±)-2-phenylbutyric acid (**G**), and (*S*)-(–)-3-phenyllactic acid (**H**), along with indole-3-acetic acid (IAA) (**I**) as positive control, were tested on *B. plantarii*-impregnated gellan plates, in which Winogradsky’s mineral mixture supplemented with 50 g L^−1^ sucrose, 500 mg L^−1^ yeast extract, and 0.1 mM Fe_2_(SO_4_)_3_ was solidified with 10 g L^−1^ gellan gum. An MeOH solution of each test compound prepared at 0.1, 1, 10, and 100 mM was loaded on a paper disk for the assay, and the absolute amount of the test compound on each paper disc is shown in a sub-table in the panel. The sub-number shows the absolute amount of the compound loaded (e.g. A2 is 3-pyridylacetic acid ·HCl at 100 nmol). CT is the control (MeOH only). Red arrows on the plate photograph indicate tropolone inhibition zone.

In the paper disk assay using PD agar medium impregnated with *B. plantarii* cells, PAA analogues were loaded on a paper disk as 10 μL of each 1 μM solution or the respective diluted solutions. Among the PAA analogues tested, *p*-tolylacetic acid, (*R*)-(–)-2-methylphenylpropionic acid, and (*p*-isopropylphenyl)acetic acid inhibited tropolone production as effectively as IAA at the same concentration (Fig. 6).

## Discussion

IAA is a well-known pro-growth plant phytohormone that induces cell division and lateral root development^24^. Here, we show that IAA is a non-antibacterial diffusible component that blocks tropolone synthesis and suppresses rice seedling blight disease. This finding reveals that IAA is not simply a hormone but actually a key mediator between plant and tropolone-producing *B. plantarii* to regulate virulence of the phytopathogen. This may be the mechanism by which indole suppresses shigatoxin production in *Escherichia coli*^25^.

Based on a report on tropone biosynthesis in the marine bacterium *Phaeobacter gallaeciensis,* class *Alphaproteobacteria*^26^, we investigated the mechanism by which IAA interferes with tropolone production. PAA is an aromatic metabolite that is produced when *B. plantarii* are fed L-phenylalanine, and it accumulates in the culture fluid^27^. PAA production by *B. plantarii* is thus directly linked to tropolone production, and levels of PAA were particularly elevated in PD broth cultures in the present study (Fig. 5). The main carbon source in PD broth medium is glucose, whereas the nitrogen source is a mixture of amino acids primarily composed of non-aromatic L-leucine, L-aspartic acid, L-glutamic acid, and L-glutamine; only a minor amount of aromatic amino acids, i.e., L-phenylalanine and L-tyrosine, is present^28^,^29^. We also confirmed production of PAA by *B. plantarii* in minimum salt medium^9^ (data not shown). Therefore, we attribute the high productivity of PAA in *B. plantarii* to an active shikimate pathway^30^ along with decarboxylation-coupled deamination of exogenous L-phenylalanine^31^, suggesting that PAA is the direct precursor of tropolone.

Feeding experiments using deuterium-labelled L-phenylalanine showed that L-phenylalanine is effectively converted to not only deuterium-labelled PAA but also deuterium-labelled tropolone (Fig. 4). Furthermore, direct feeding of deuterium-labelled PAA to *B. plantarii* led to incorporation of deuterium on the phenyl group into the tropolone seven-membered ring (Figure S6). Thus, tropolone biosynthesis in *B. plantarii* involves bioconversion of PAA to tropolone via an aromatic ring-rearrangement. This process is similar to the steps in the biosynthetic pathway of a tropolone-type metabolite, tropodithietic acid, in *P. gallaeciensis* (e.g., transamination and oxidative decarboxylation of phenylalanine to PAA)^26^. PAA production in *P. gallaeciensis* was not observed when it was cultured in medium containing no L-phenylalanine, suggesting that L-phenylalanine is the most important precursor of PAA in *P. gallaeciensis*^30^. The reason why four but not five carbons were labelled in tropolone converted from [*ring*-^2^H_5_]-PAA remains unclear; however, the mechanisms of ring rearrangement should be investigated in future studies by using ^13^C-labeled PAA at the C2-position.

Exposure of *B. plantarii* to IAA during the exponential phase of culture growth suppressed PAA production, whereas IAA more effectively decreased PAA-derived tropolone production (Fig. 5). IAA is structurally analogous to PAA and acts as a competitive inhibitor mainly against ring rearrangement on PAA for conversion into tropolone. IAA produced by the potent antagonistic bacterium *B. heleia* against bacterial rice blight disease thus demonstrated disturbance of virulence-associated phytotoxin production by the bacterial pathogen without any antibacterial mode of action. These data suggest that IAA-mediated biological and chemical control strategies against virulence of *B. plantarii* via tropolone production could attenuate symptom development and restore growth and extension of the roots. Similarly, IAA is known as the mediator of attenuation of potato tuber lesions by *Fusarium solani* f. sp. *eumartii,* where activity of the chymotrypsin-like *Fusarium* extracellular serine protease was shown to be dose-dependently inhibited by exogenous IAA without any fungal growth repression^32^.

In summary, the insight that we have provided into microbial interspecies interaction in the rhizosphere ecosystem may lead to the development of novel biocontrol agents. Such agents may reduce the symptoms caused by phytopathogens while allowing them to survive and contribute to a well-balanced rhizosphere ecosystem.

## Methods

### Analytical instruments and chemicals

Primary analytical instruments and chemicals used were as follows: Waters 600 HPLC (Waters, MA, USA) with an L-column2 ODS column (250 mm × i.d. 4.6 mm; particle size 5 μm); Agilent 218 Purification Systems (Agilent, Santa Clara, CA, USA) with a Prep-C18 column (50 mm × i.d. 30 mm; particle size 5 μm); Agilent 7890A GC (Agilent, CA, USA) with an HP-5 capillary column (30 m × i.d. 0.25 mm); MS spectrometers JEOL JMS-T100GCV, JMS-SX-102 (JEOL, Tokyo, Japan) and Agilent 7000C GC-MS/MS (Santa Clara, CA, USA) with an Agilent HP-5 glass capillary column (Agilent Technologies, 30 m × i.d. 0.32 mm) under the conditions of initial temperature of 100 °C for 1.5 min and heating at a rate of 60 °C min^−1^ until 300 °C with a carrier gas of He; NMR spectrometers JEOL JNM-EX270 (JEOL, Tokyo, Japan) and Bruker AM 500 (Bruker, Bremen, Germany); ABI Prism 310 Genetic Analyzer (Applied Biosystems, CA, USA); StepOnePlus Real-Time PCR thermal cycling block (Applied Biosystems, CA, USA); trifluoromethanesulfonic acid-*d* (TfOD, Energy Chemical, Shanghai, China); L-phenylalanine (TCI, Tokyo, Japan); L-phenylalanine-[*ring*-^2^H_5_] (Sigma-Aldrich, MO, USA); phenylacetic acid (PAA, Sigma-Aldrich, St. Luis, MO, USA). Other chemicals used for preliminary screenings in the present study were purchased from TCI and Wako (Osaka, Japan).

### Bacterial strains and culture conditions

*B. plantarii* was kindly provided by Professor Yuichi Takikawa (Faculty of Agriculture, Shizuoka University) and Kumiai Chemical Industry Co. Ltd. (Tokyo, Japan). Thirteen bacterial species previously screened from the rice rhizosphere as tolerable to 10 mM catechol^7^ were tested for biocontrol efficacy against rice seedling blight. All of the bacterial species were routinely grown at 25 °C in the dark in potato dextrose (PD) broth (pH 6.2) either statically or with agitation at 110 rpm. Alternatively, cultures were grown on a PD agar plate that was solidified with 1.5% agar (Wako, Osaka, Japan).

### Biocontrol test against bacterial rice seedling blight

Seeds of rice (*Oryza sativa* var. *japonica* cv. Koshihikari) surface-sterilized with 0.5% hypochlorite and 70% ethanol were subjected to inoculation with *B. plantarii* by means of soaking in 10 mL of *B. plantarii* cell suspension (10^3^ colony forming units (CFU) mL^−1^) in Petri dishes. For treatment with test bacteria, the seeds were simultaneously inoculated with 50 μL of the bacterial cell suspension (10^6^ CFU mL^−1^) and antagonistic efficacy was evaluated. Surface-sterilized rice seeds infested with *B. plantarii* only (control) or the testing bacterium only were also prepared along with those that were not inoculated with bacteria. All of the rice seeds were incubated, transplanted, and subsequently cultivated as previously described^10^. After 5 d of cultivation, the length of shoots and roots and the appearance or abrogation of symptoms such as discoloration of the shoot were measured.

### Metabolic changes of *B. plantarii* following co*-*culture with *B. heleia* PAK1-2

*B. heleia* PAK1-2 was point-inoculated at the centre of a PD agar plate impregnated with *B. plantarii* cells as previously described^10^. After 3-d incubation, colony morphology was observed under a light microscope (Olympus IX70, Tokyo, Japan). Plain PD agar plates point-inoculated with *B. plantarii* only or *B. heleia* only were used as controls. PD broth medium inoculated with *B. plantarii* and *B. heleia* PAK1–2 (both at 10^3^ CFU mL^−1^) or *B. plantarii* only (for control) was quantitatively monitored for tropolone production. After 72-h-shake-culturing, the culture fluid was subjected to solid-phase extraction and HPLC.

### Tropolone production assay using *B. plantarii*-cell-impregnated agar plates

PD-agar medium containing 0.1 mM FeSO_4_ and viable cells of *B. plantarii* were prepared for bioassay to identify tropolone production inhibitors as follows: PD broth medium supplemented with 1.0% agar was autoclaved at 120 °C for 20 min and cooled to below 50 °C; then, 1 mL of 10 mM FeSO_4_ solution filtered through a sterile membrane (0.4 μm cellulose acetate, Millipore, Darmstadt, Germany) was added and mixed well. The liquefied PD agar mixed well with the cell suspension of pre-cultured *Burkholderia plantarii* (10 mL of bacterial cell suspension as 10^6^CFU mL^−1^) was then cast on plastic Petri plates (Bio-Bik, Ina-Optika Co. Ltd, Osaka, Japan). This agar medium impregnated with *B. plantarii* cells was used in the screening assay for inhibitors against production of tropolone. For a more sensitive assay, Winogradsky’s medium-based gellan plate (50 g L^−1^ sucrose, 0.5 g L^−1^ yeast extract, 10 g L^−1^ gellan gum) containing 0.1 mM FeSO_4_ was used instead.

A paper disk with a diameter of 9 mm and thickness of 0.5 mm, on which a solution of a test compound was loaded, was placed on a 9-cm sterile Petri plate containing 10 mL of medium. In the test, paper disks that had absorbed 10 μL of a test compound solution at each concentration and then been air-dried were placed on the assay plates followed by incubation at 28 °C for 5 days. When the test compounds showed inhibitory activity against tropolone production observed as tiny but visible particles of dark reddish precipitated crystals, the assay plate around the paper disc was visible as a turbid background area without any dark crystalline particles of tropolone because *B. plantarii* cell growth was permitted.

### Metabolic profiling of *B. plantarii* using ^1^H-NMR, GC and mass spectroscopy

*B. plantarii* was inoculated into 50 mL of PD broth medium (10^3^ CFU mL^−1^) and shake-cultured for 24 h at 25 °C. After removal of bacterial cells, the culture fluid was adjusted to pH 3.5 with 2 M sulphuric acid and extracted twice with 50 mL of EtOAc. The organic layer was dried over anhydrous Na_2_SO_4_, concentrated, and re-dissolved in EtOAc for metabolic profiling according to the method described by Clarke and Haselden^22^.

A half portion of the EtOAc solute was concentrated and re-dissolved in methanol-*d*_4_ for ^1^H-NMR analysis without any purification process. The other half of the EtOAc solute was further concentrated to 100 μL, of which 0.2 μL was subjected to GC-MS analysis using Agilent 7890A GC in combination with JMS-SX-102 MS spectrometer to obtain a GC profile of total ion chromatography. The EI fragmentation pattern of the most dominant chemical component was recorded by the GC-MS analysis. In parallel, the concentrated sample of the crude mixture was directly applied for FI-MS and EI-MS analyses.

### Isolation and identification of the *B. heleia* PAK1-2-derived compound IAA that represses tropolone production in *B. plantarii*

To collect secondary metabolites produced by *B. heleia* PAK1-2, 3 mL of *B. heleia* PAK1-2 cell suspension (10^6^ CFU mL^−1^) was inoculated into a 3-L culture of PDB and shake-cultured for 3 d at 25 °C in the dark. Bacterial cells were separated by centrifugation at 8 500 × *g* for 10 min. The culture fluid was passed through Cosmosil 75C18-OPN (250 g, Nacalai Tesque, Kyoto, Japan) on a Buchner funnel previously conditioned with MeOH (2 L) followed by an excess of Milli-Q water (3 L). After removal of the void water by vacuum, the substances trapped by the Cosmosil particles were eluted with a sufficient volume of MeOH (2 L). The eluates (318 mg) were concentrated and suspended in chloroform; then, they were subjected to silica gel column chromatography (50 g, GF_60_ 35 to 70 mesh, Merck, Darmstadt, Germany) by stepwise elution with 1% to 100% MeOH in chloroform to obtain 10 fractions. Fractions exhibiting similar thin-layer chromatography profiles were combined to obtain three main fractions (fractions 1, 2, and 3 for 5–20%, 25–50%, and 60–100% MeOH/chloroform respectively). To search for active principles that repressed tropolone production, these fractionated metabolites were subjected to an agar diffusion assay on *B. plantarii*-impregnated PD agar containing 0.1 mM FeCl_3_^10^. Physicochemical properties of the active principle isolated from fraction 2 are shown in Table S1.

### Identification and quantification of phenyl acetic acid produced by *B. plantarii*

Phenylacetic acid (PAA) was identified as the main metabolite of *B. plantarii* in the early growth stage (Fig. 2). Cultures of *B. plantarii* in PD broth medium were sampled at 0, 6, 12, 18, 24, 30, 36, 42, 48, 60 and 72 h in a time-course experiment. Each culture was sampled (2 mL) in triplicate and centrifuged at 8 500 × *g* for 10 min, and then the resulting supernatant (1.5 mL) was then subjected to solid phase extraction with a preconditioned C18 cartridge of 200 mg resin (Sep-Pak Vac 3cc, Waters, Milford, MA, USA). The resulting cartridge column was washed with water (3 mL), the water was voided, and then the column was eluted with methanol (3 mL). The methanolic elutes were concentrated and re-dissolved in 150 μL of methanol, of which 10 μL was injected into an HPLC system with an isocratic mobile phase of MeOH:10 mM phosphoric acid (85:15) and a photodiode array detector (at 260 nm). The retention time (*tR*) of PAA was 3.9 min. Standard solutions (10 μL) of PAA (0.01, 0.1, 0.5, 1 and 10 mM) were also subjected to HPLC for preparation of a PAA standard curve. This yielded an equation of *y* = 0.0001*x*–0.216 (*R^2^* = 0.996). *y* = concentration of PAA (mM), *x* = absolute peak intensity of PAA.

A conventional feeding experiment for tropolone production was performed for *B. plantarii* using PD broth media supplemented with L-phenylalanine at 0.5, 1, and 2 mM in triplicate at each concentration, and PD broth media containing exogenous PAA at 0.5, 1, and 2 mM were run in triplicate. After 36 h of shake-culture, each culture medium was also subjected to HPLC analysis for quantification of tropolone production.

### Deuterium-labelled precursors and feeding experiments

Additional feeding experiments were performed using deuterium-labelled substrate candidates. *B. plantarii* was shake-cultured in 50 mL of PD broth medium supplemented with 2 mM commercially available L-phenylalanine-[*ring*-^2^H_5_]. EtOAc extracts from the 36-h-shake-cultured fluid were subjected to FI-MS analysis (JEOL JMS-T100GCV) without further purification.

Deuterium-labelled PAA was obtained by treatment of authentic PAA (500 mg) with TfOD (6.5 mL, 20 equivalents) at room temperature for 12 h according to a previously reported approach^33^. The reaction mixture was gently diluted in 50 mL of Milli-Q water and subjected to liquid-liquid partition with chloroform (50 mL, twice). The organic layer was dried with anhydrous Na_2_SO_4_, concentrated, re-dissolved in MeOH and purified by Agilent 218 preparative HPLC to yield PAA-[*ring*-^2^H_2_-_5_] (419 mg) (Fig. 4). The mixture of deuterated PAA was further added to PD broth media at ~1.4 mM (200 mg L^−1^) for shake-culture of *B. plantarii*. The culture fluid after 36 h of incubation was extracted with EtOAc; concentrated substances were re-dissolved in EtOAc (100 μL) and subjected to FI-MS and GC-MS/MS (in 1 μL portions) for detection of deuterated tropolone and qualitative analysis for the deuteration ratio.

### Gene expression analysis using quantitative real time-PCR for *plaI* and *plaR* genes associated with the AHLs-QS system

To investigate the mechanism by which tropolone production is repressed by IAA, we examined the expression of QS-associated genes, particularly those related with AHL. To this end, *B. plantarii* was shake-cultured for 12 h in plain PD broth medium (control) or PD broth that contained IAA (200 μM) and examined for expression of *plaI* (AHLs synthase gene) and *plaR* (AHLs receptor gene) using real-time quantitative reverse transcription PCR (qRT-PCR). Isolation of total RNA, digestion of the remaining genomic DNA, cDNA synthesis, and removal of remaining RNA were sequentially conducted as reported previously^10^, and qRT-PCR was performed in a StepOnePlus Real-Time PCR thermal cycling block (Applied Biosystems, CA, USA). Specific-primers for the qRT-PCR were *plaI RT-forward* (5′-GGA AGA CGA AAA ATT CGA G-3′)/*plaI RT-reverse* (5′-TAC ACC GGT ATC GTC G-3′); *plaR RT-forward* (5′-GAG ATC AAC AGC CTG AC-3′)/*plaR RT-reverse* (5′-AGC GAA TGC GAG AGA T-3′); and *rpoD* RT-forward (5′-CTA CAA GTC GAA GTC CTA C-3′)/*rpoD RT-reverse* (5′-ATC GAC ATC AGT TCG TTC-3′). An initial step of 30 s at 95 °C followed by 30 cycles of 5 s at 95 °C, 30 s at 52 °C, and 1 min at 72 °C w performed. Fold changes in the expression of each target gene was calculated in comparison to those in the internal control gene *rpoD* (RNA polymerase sigma factor RpoD) according to the 2^−ΔΔ*C*T^ method^34^.

### Statistical analyses

Statistical analysis was performed using Microsoft Excel 2012 for Student’s *t* and Student-Newman-Keuls tests with appropriate calculation of statistically significant differences as indicated. *P* < 0.05 was considered significant.

## Additional Information

**How to cite this article**: Wang, M. *et al.* Indole-3-Acetic Acid Produced by *Burkholderia heleia* Acts as a Phenylacetic Acid Antagonist to Disrupt Tropolone Biosynthesis in *Burkholderia plantarii. Sci. Rep.* **6**, 22596; doi: 10.1038/srep22596 (2016).

## Supplementary Material

Supplementary Information
