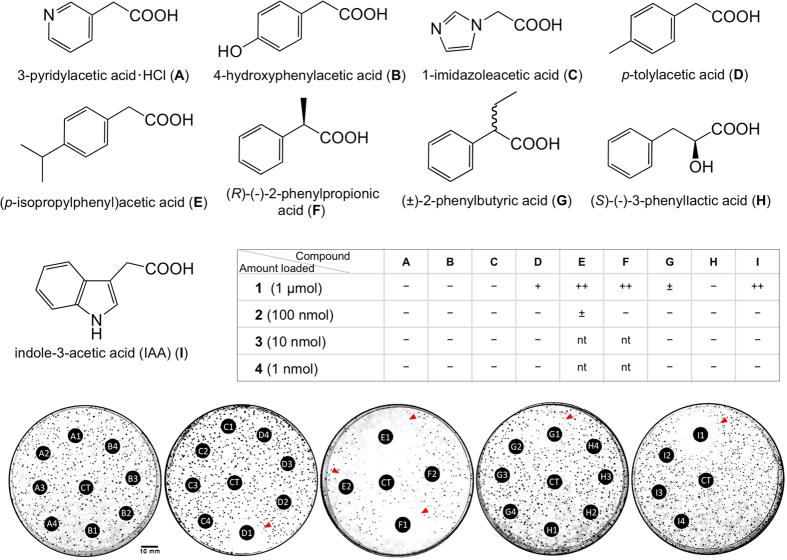# Corrigendum: Indole-3-Acetic Acid Produced by *Burkholderia heleia* Acts as a Phenylacetic Acid Antagonist to Disrupt Tropolone Biosynthesis in *Burkholderia plantarii*

**DOI:** 10.1038/srep26217

**Published:** 2016-05-20

**Authors:** Mengcen Wang, Seiji Tachibana, Yuta Murai, Li Li, Sharon Yu Ling Lau, Mengchao Cao, Guonian Zhu, Makoto Hashimoto, Yasuyuki Hashidoko

Scientific Reports 6: Article number: 22596; 10.1038/srep22596 published online: 03032016; updated: 05202016.

In this Article, the compound ‘(*R*)-(–)-2-phenylpropionic acid’ was incorrectly given as ‘(*R*)-(–)-2-methylphenylpropionic acid’. In the Results section under subheading ‘Interference of the tropolone biosynthetic pathway of *B. plantarii* by PAA analogues’,

“Among the PAA analogues tested, *p*-tolylacetic acid, (*R*)-(–)-2-methylphenylpropionic acid, and (*p*-isopropylphenyl)acetic acid inhibited tropolone production as effectively as IAA at the same concentration (Figure 6).”

should read:

“Among the PAA analogues tested, *p*-tolylacetic acid, ‘(*R*)-(–)-2-phenylpropionic acid, and (*p*-isopropylphenyl)acetic acid inhibited tropolone production as effectively as IAA at the same concentration (Figure 6).”

The correct Figure 6 and its accompanying legend appear below as Figure 1.

Eight compounds, i.e., 3-pyridylacetic acid HCl (**A**), 4-hydroxyphenylacetic acid (**B**), 1-imidazoleacetic acid (**C**), *p*-tolylacetic acid (**D**), (*p*-isopropylphenyl)acetic acid (**E**), (*R*)-(–)-2-phenylpropionic acid (**F**), (±)-2-phenylbutyric acid (**G**), and (*S*)-(–)-3-phenyllactic acid (**H**), along with indole-3-acetic acid (IAA) (**I**) as positive control, were tested on *B. plantarii*-impregnated gellan plates, in which Winogradsky’s mineral mixture supplemented with 50 g L^−1^ sucrose, 500 mg L^−1^ yeast extract, and 0.1 mM Fe_2_(SO_4_)_3_ was solidified with 10 g L^−1^ gellan gum. An MeOH solution of each test compound prepared at 0.1, 1, 10, and 100 mM was loaded on a paper disk for the assay, and the absolute amount of the test compound on each paper disc is shown in a sub-table in the panel. The sub-number shows the absolute amount of the compound loaded (e.g. A2 is 3-pyridylacetic acid ·HCl at 100 nmol). CT is the control (MeOH only). Red arrows on the plate photograph indicate tropolone inhibition zone.

## Figures and Tables

**Figure 1:**