# Supplementary material for: Indole-3-Acetic Acid Produced by Burkholderia heleia Acts as a Phenylacetic Acid Antagonist to Disrupt Tropolone Biosynthesis in Burkholderia plantarii
Source: Sci Rep. 2016 Mar 3;6:22596. doi: 10.1038/srep22596 (PMC4776283; doi:10.1038/srep22596)
Supplement: Supplementary Information [file srep22596-s1.pdf]

Indole-3-Acetic Acid Produced by *Burkholderia heleia* Acts as a Phenylacetic Acid Antagonist to Disrupt Tropolone Biosynthesis in *Burkholderia plantarii*

Mengcen Wang [1,2]
Seiji Tachibana [1]
Yuta Murai [1,3]
Li Li [1]
Sharon Yu Ling Lau [1]
Mengchao Cao [2]
Guonian Zhu [2]
Makoto Hashimoto [1]
Yasuyuki Hashidoko [1]

[1] Research Faculty of Agriculture, Hokkaido University, Kita 9, Nishi 9, Kita-ku, Sapporo 060-8589, Japan.

[2] Institute of Pesticide and Environmental Toxicology, Zhejiang University, No. 268 Kaixuan Road, Hangzhou 310029, China.

[3] Frontier Research Center for Post-Genome Science and Technology, Faculty of Advanced Life Sciences, Hokkaido University, Kita 10, Nishi 8, Kita-ku, Sapporo 060-0810, Japan.

Table S1. Isolation and identification of indole-3-acetic acid as *B. heleia* PAK1-2-derived active compound that represses tropolone production in *B. plantarii*

δ_H (multiplicity, *J* in Hz)	δ_C (multiplicity)	C-H substitution
—	C_{11}, 176.22 (C)	**COOH**
—	C_8, 137.67 (C)	(NH)**C**R=C
—	C_9, 128.31 (C)	R_2**C**=C
7.15 (1H, *s*)	C_2, 124.25 (CH)	**CH**
7.09 (1H, *t*, *J*=8)	C_6, 122.06 (CH)	**CH**
7.01 (1H, *t*, *J*=8)	C_5, 119.44 (CH)	**CH**
7.53 (1H, *d*, *J*=8)	C_4, 119.06 (CH)	**CH**
7.33 (1H, *d*, *J*=8)	C_7, 111.84 (CH)	**CH**
—	C_3, 108.50 (C)	R_2**C**=C
3.72 (*br s*, 2H)	C_{10}, 31.39 (CH_2)	CH_2
NH	—	—

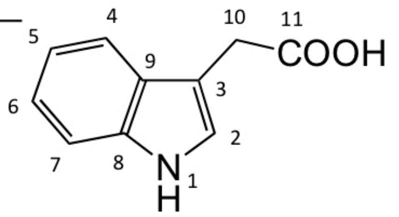

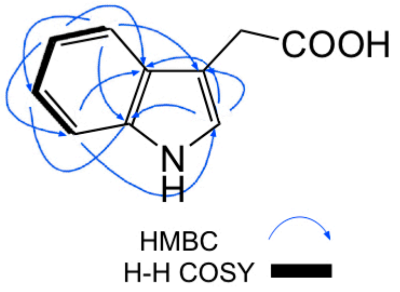

The major component repressing tropolone production in fraction 2 (25-50% MeOH) was obtained as a colourless powder (21 mg) with preparative thin-layer chromatography to give spectroscopic data. FD-MS (*m/z*, rel. int. %): 175.1 (100, $[M]^+$); FD-HR-MS: found 175.0615 ($C_{10}H_9NO_2$, calcd. 175.0633); EI-MS (*m/z*, rel. int. %): 175 (14, $[M]^+$), 130 (100, $[M\text{-}COOH]^+$), 103 (10), and 77 (14). ^{1}H-NMR (NON, H-H COSY) and ^{13}C-NMR (BCM, DEPT, HMQC and HMBC) spectra of the tropolone production-repressing principle from *B. heleia* PAK1-2 was measured in methanol-d_4 to be assigned it as indole-3-acetic acid (IAA). Multiplicities of proton and carbon are deduced from DEPT and HMQC data, while C-H correlations including the partial structures are deduced from HMQC.

Figure S1. Phylogenetic analysis of the 16S rRNA gene of genus *Burkholderia* and isolate PAK1-2 using neighbor-joining tree

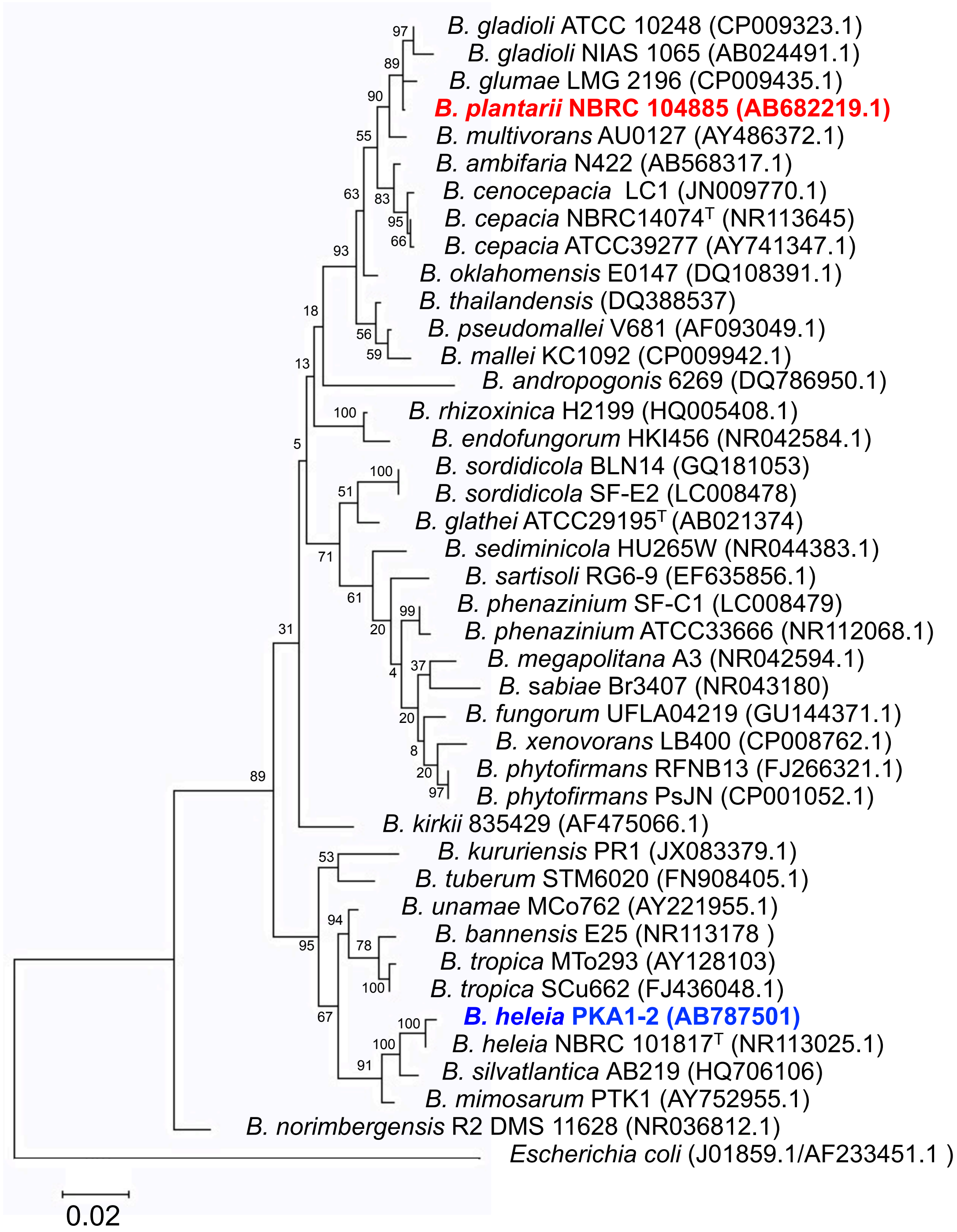

The sequence of the 1.4-kbp 16S rRNA gene region (from 79 to 1464 for *Escherichia coli* 16S ribosomal RNA) from isolate PKA1-2 was subjected to phylogenetic analysis among representative members of genus *Burkholderia* at the same region using MEGA 6.06 (Wineskin). Multiple alignments of the data collected from NCBI database were run using Clustal W and the phylogenetic tree was constructed using neighbour-joining methods with 1000 bootstrap replicates. Bar, 0.02 nucleotide substitutions per nucleotide site. *E. coli* is used as an outgroup. Note that *B. plantarii* and *B. heleia* are relatively far from each other among the members of genus *Burkholderia*.

Figure S2. Colony outgrowth of *B. plantarii* cultured with *B. heleia* PAK1-2

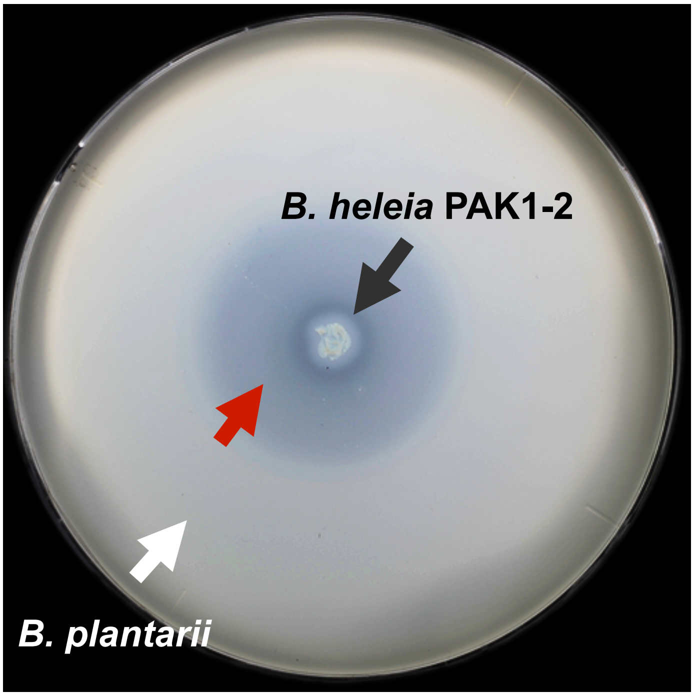

Inhibitory effect of *B. heleia* PAK1-2 was observed on a *B. plantarii*-impregnated plate: white arrow indicates the growth background of *B. plantarii* and black arrow indicates the growth of *B. heleia* PAK1-2 that were point-inoculated in the center of the plate, while the red arrow indicates the halo due to inhibition of *B. plantarii* growth.

Figure S3. Gene expression analysis of AHLs-QS system in *B. plantarii* exposed to IAA by real time qRT-PCR

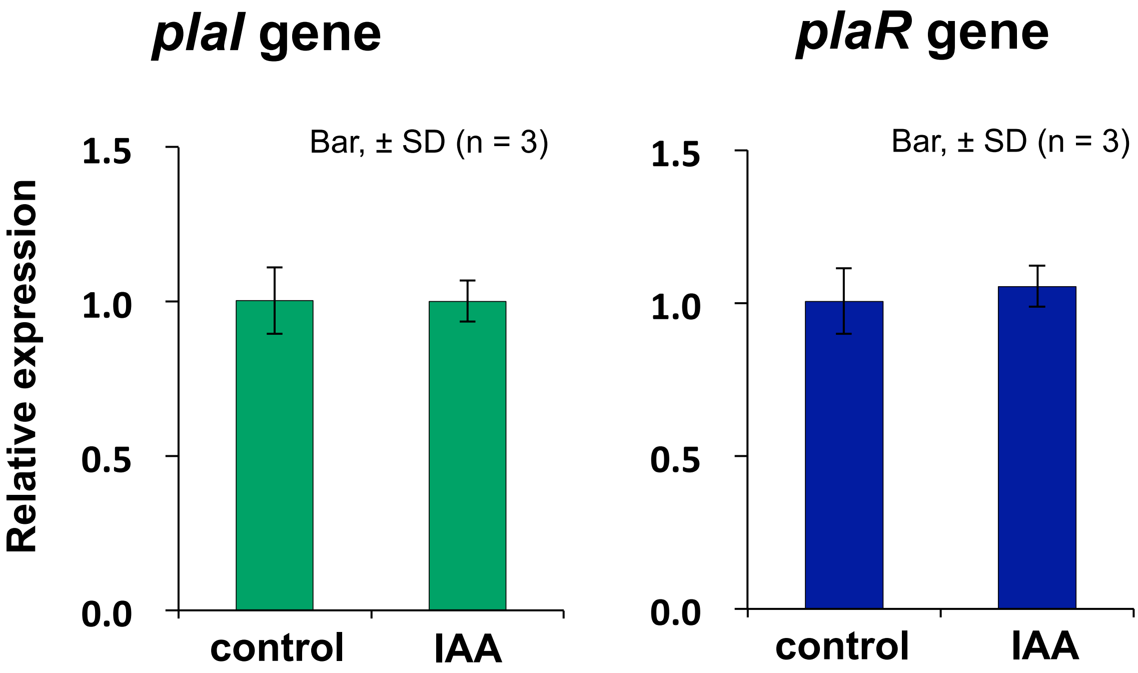

Expression of *plaI* and *plaR* genes in *B. plantarii* was not significantly inhibited by IAA in contrast to control ($P > 0.05$). Values are means ± SD (shown by error bars) ($n = 3$).

Figure S4. Metabolic profile of *B. plantarii* and characterization of dominant metabolite by GC-MS

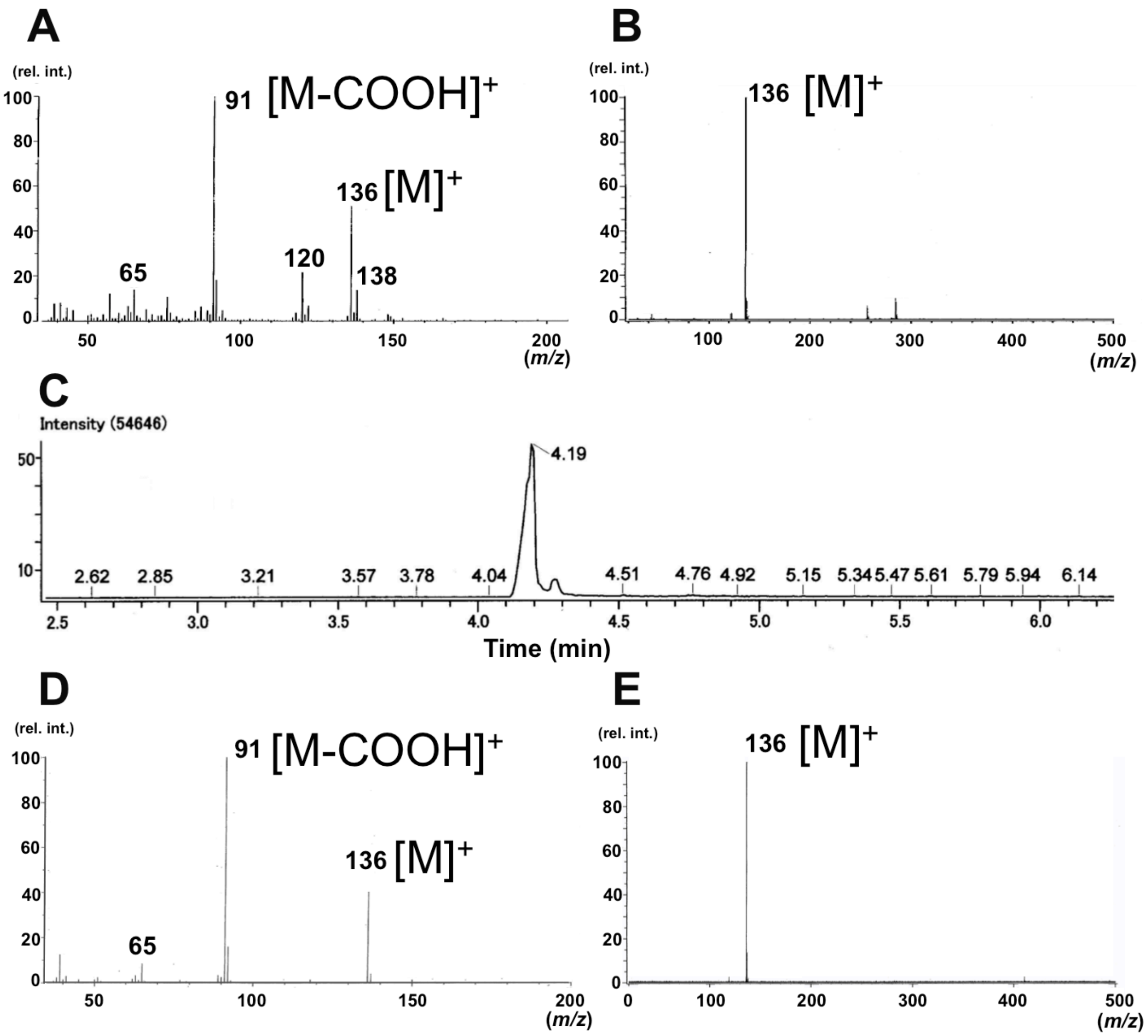

Direct EI-MS (A) and FI-MS (B) spectra of a mixture of metabolites extracted with EtOAc from culture fluid of *B. plantarii* at early culturing stage (24 h). An ion monitoring profile of the mixture in GC-MS (C). EI-MS (D) and FI-MS (E) spectra of the dominant metabolite detected at *t*R 4.19 min by GC-MS.

Figure S5. ^{1}H-NMR spectroscopic analysis for a mixture of EtOAc soluble secondary metabolites by *B. plantarii*

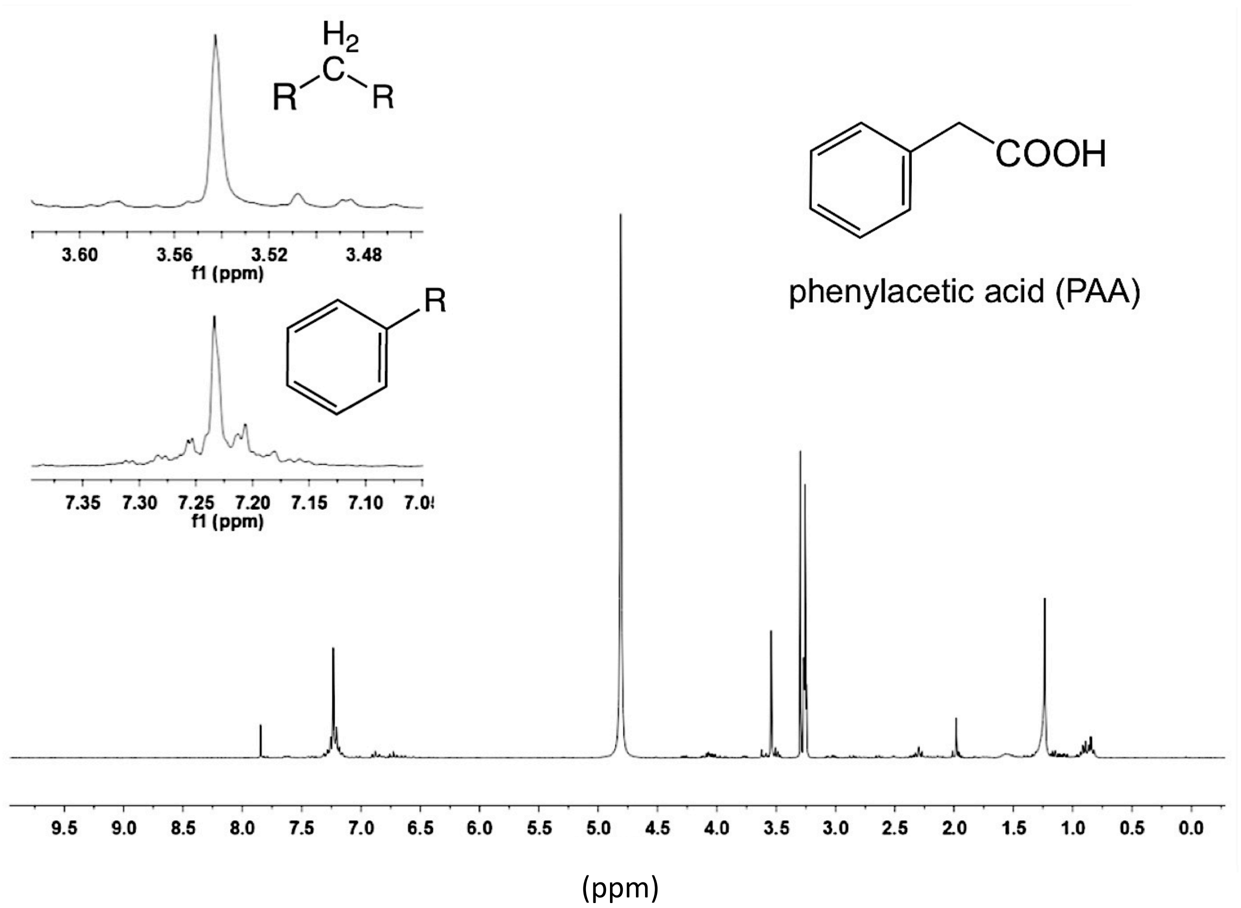

A portion of the EtOAc solute (2.5 ml) was concentrated and re-dissolved in methanol-d_4 for ^{1}H-NMR analysis without any purification process. In ^{1}H-NMR spectrum of the mixture, major constituent was structurally identical to phenylacetic acid (PAA).

Figure S6. Identification of isotopomeric tropolones from deuterated PAA by GC-MS/MS

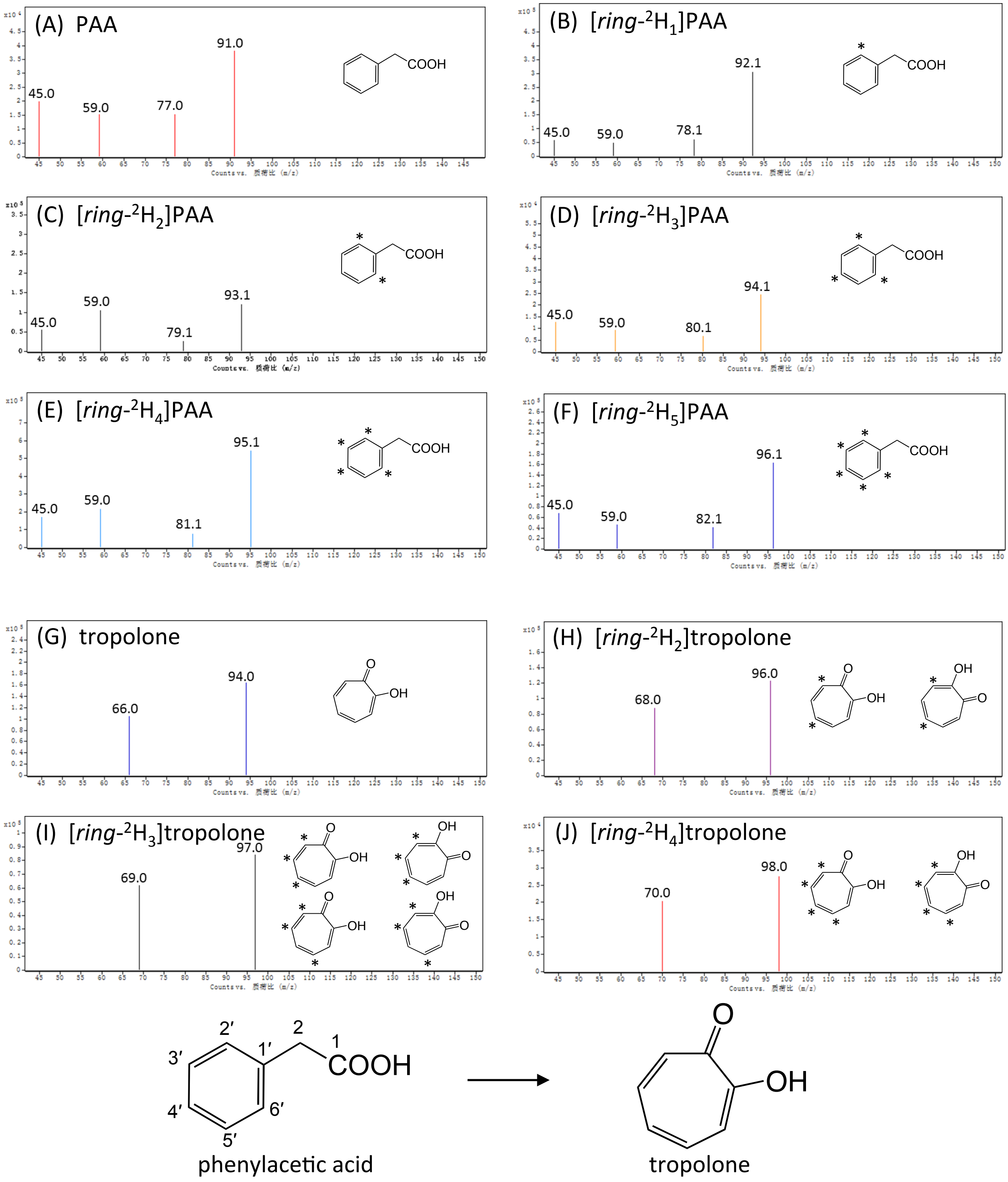

Deuterated PAA synthesized was quantified using GC-MS/MS in comparison with authentic PAA (A); this revealed that the benzene ring was substituted by 1 to 5 of deuterium atoms (B-F). Also authentic tropolone (G) was compared with deuterated tropolones converted by *B. plantarii* from these deuterated PAAs. In the GC-MS/MS, deuterated tropolones maintained 2 to 4 deuterium atoms on the tropolone ring (H-J).

Figure S7. Effect of indole and its derivatives on phenylacetic acid and tropolone production by *B. plantarii*

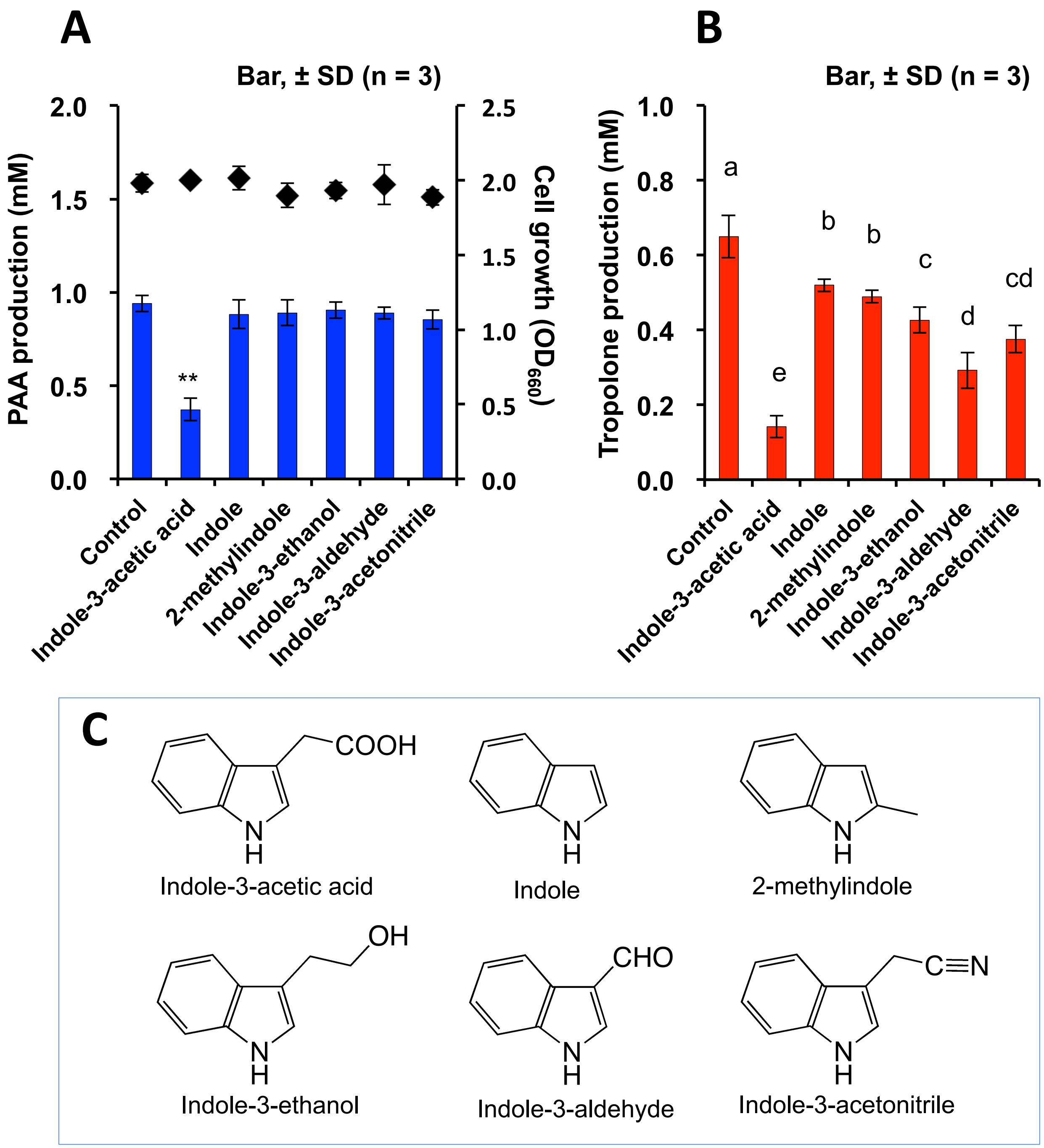

PAA production by *B. plantarii* following exposure to IAA and other indole derivatives (columns) and cell growth (plots, ◆) quantified from *B. plantarii* PD broth cultures containing 200 μM indole derivative (A). ** *P*< 0.01 by Student's-*t* test. Tropolone production by *B. plantarii* after exposure to IAA (B). Diverse alphabet indicates significant difference as determined by Student-Newman-Keuls test. Values (mM) in graphs A and B are means ± SD (shown by error bars). Chemical structures of indole derivatives tested are shown in panel C.
